# Ionizing Radiation-Induced Cellular Senescence in Normal, Non-transformed Cells and the Involved DNA Damage Response: A Mini Review

**DOI:** 10.3389/fphar.2018.00522

**Published:** 2018-05-22

**Authors:** Mengqian Li, Liting You, Jianxin Xue, You Lu

**Affiliations:** Department of Thoracic Oncology, Cancer Center, West China Hospital, Sichuan University, Chengdu, China

**Keywords:** cellular senescence, ionizing radiation, DNA damage, cell cycle, senolytic

## Abstract

Cellular senescence is identified by a living cell in irreversible and persistent cell cycle arrest in response to various cellular stresses. Senescent cells secrete senescence-associated secretory phenotype factors that can amplify cellular senescence and alter the microenvironments. Radiotherapy, via ionizing radiation, serves as an effective treatment for local tumor control with side effects on normal cells, which can induce inflammation and fibrosis in irradiated and nearby regions. Research has revealed that senescent phenotype is observable in irradiated organs. This process starts with DNA damage mediated by radiation, after which a G2 arrest occurs in virtually all eukaryotic cells and a mitotic bypass is possibly necessary to ultimately establish cellular senescence. Within this complex DNA damage response signaling network, ataxia telangiectasia-mutated protein, p53, and p21 stand out as the crucial mediators. Senolytic agents, a class of small molecules that can selectively kill senescent cells, hold great potential to substantially reduce the side effects caused by radiotherapy while reasonably steer clear of carcinogenesis.

## Introduction

For the first time, Hayflick and Moorhead employed the word “senescence” to define the finite capacity of a cell strain’s *in vitro* lifespan in Hayflick and Moorhead (1961). In contemporary literature, the “senescence” state of cells is hereby designated as cellular senescence, and cells experiencing cellular senescence are senescent cells (SNCs). Generally, SNCs are identified by the permanent loss of proliferative potential, the expression of SA-β-gal (displaying the highest enzymatic activity at pH 6.0), and the upregulated cyclin-dependent kinase inhibitors, such as p21 and p16 (Childs et al., 2017). They are relatively resistant to apoptosis (Marmary et al., 2016) and can be obliterated by the immune responses (Xue et al., 2007; Tchkonia et al., 2013). Notably, SNCs are not quiescent but are metabolically active, which grow in cell mass (size or volume) and secrete SASP factors that will be elaborated on thereinafter (Krizhanovsky et al., 2008; Leontieva and Blagosklonny, 2010; Childs et al., 2017). Nevertheless, SNCs cannot grow in size indefinitely, but they may restrain growth by self-degradation via lysosomal enzymes leakage, such as the aforementioned SA-β-gal (Demidenko and Blagosklonny, 2008). Possibly, persistent and irreversible SNCs may adopt some forms of cell death, such as apoptosis, which is not a primary response to the senescence-inducing treatment (Gewirtz et al., 2016). The most intriguing phenotype is that SNCs may be equipped with overabundance of cyclin D1 in combination with activated p53-p21 and/or p16-Rb signaling pathway (Dulic et al., 1993; Lien et al., 2004; Saegusa et al., 2004; Demidenko and Blagosklonny, 2008; Leontieva et al., 2013).

A growing body of evidence supports that the upregulation of cellular senescence levels underlies organismal senescence, while using senolytic agents to selectively induce death in SNCs improves organ function (Baker et al., 2016; Hashimoto et al., 2016; Pan et al., 2017; Schafer et al., 2017) (**Table 1**). A senolytic (from the words “senescence” and “lytic”) agent belongs to the class of small molecules that can selectively kill senescent cells (Childs et al., 2015).

**Table 1:** A summary of the first publication of important small molecule senolytic agents (small molecules that can selectively kill senescent cells).

Name of the senolytic agents	Targeted molecular	Major studied types of cell/organ/animal	Reference
AP20187	p16^INK4A^ expressing adipocytes	Transgenic INK-ATTAC progeroid mice (*in vivo*)	Baker et al., 2011
Rapamycin or sirolimus	mTOR complex mTORC1	(I) Partly suppressed the SASP, especially IL-1α in IR-induced SNCs (*in vitro*) (II) Repressed the proliferation of senescent tumor cells after subcutaneous implantation (*in vivo*)	Laberge et al., 2015
siRNA	EFNB1 or 3, PI3KCD, p21, BCL-xL, PAI-2	Senescent human abdominal subcutaneous preadipocytes (*in vitro*)	Zhu et al., 2015
Dasatinib (D)/BMS-354825 and quercetin (Q)	D: multiple tyrosine kinases Q: PI3K	(I) Selectively killing of both senescent preadipocytes, endothelial cells MEFs and MSCs (*in vitro*) (II) Senile mice (>24 months-old) (*in vivo*)	
(I) Smomelotinib (CYT387) and INCB18424 (II) Ruxolitinib (INCB18424)	JAK pathway	(I) Senescent human primary. preadipocytes (*in vitro*) (II) 24-month-old C57BL/6 male mice (*in vivo*)	Xu et al., 2015
ABT-737	BCL-2, BCL-W, and BCL-XL	(I) Irradiated male mice (*in vivo*) (II) Double-transgenic K5-rtTA/tet-p14 mice	Yosef et al., 2016
ABT263 (a paralog of ABT-737)	BCL-2 and BCL-xL	Sublethally irradiated mice or normally aged mice (*in vivo*)	Chang et al., 2016
KU-60019	ATM kinase	(I) Human diploid fibroblasts, ATM-deficient GM02052 fibroblasts (*in vivo*) (II) Facilitating wound healing in aged mice (*in vivo*)	Kang et al., 2017
17-DMAG	HSP90	(I) Senescent Ercc1-/- primary MEFs II. Ercc1-/Δ mouse (model of a human progeroid syndrome)	Fuhrmann-Stroissnigg et al., 2017
Fisetin, A1331852, A1155463	Fisetin: PI3K/Akt pathway; A1331852 and A1155463: targeting BCL-XL	Specifically inducing apoptosis in SNCs. Fisetin: senescent HUVECs. A1331852 and A1155463: senescent HUVECs and IMR-90 cells	Zhu et al., 2017
FOXO4-DRI	A modified FOXO4-p53 interfering peptide	Selectively induced apoptosis of senescent cells *in vitro* and in aging mice	Baar et al., 2017

*mTOR, mammalian target of rapamycin; HSP90, heat shock protein 90; FOXO4, Forkhead box protein O4.*

Pro-senescence inducers include telomere attrition, DNA damage and mutations, enhanced oxidative stress from ROS, and the persistent response to these events within organisms (Schafer et al., 2017).

This review focuses on the medical ionizing radiation (IR)-induced cellular senescence in normal, non-transformed cells. Radiotherapy, via ionizing radiation including X-rays, γ-rays, β-particles radiation et al., is an effective treatment for controlling local tumor. When radiation energy is deposited in exposed tumors, damage occurs directly by secondary electrons and/or indirectly by ROS and harms the basic components of tumor cells, such as DNA and proteins (Hernández et al., 2015). However, inevitable damage in nearby normal regions can ignite the DNA damage response (DDR), including persistent or irreparable DNA damage, irreversible cell cycle arrest and SASP factors production, which culminates in establishing cellular senescence. In this course, the ataxia telangiectasia-mutated protein (ATM)-p53-p21 signaling pathway ranks among the most studied mechanisms to illustrate the underlying principles of cellular senescence caused by IR.

## Reactive Oxygen Species

**FIGURE 1:**
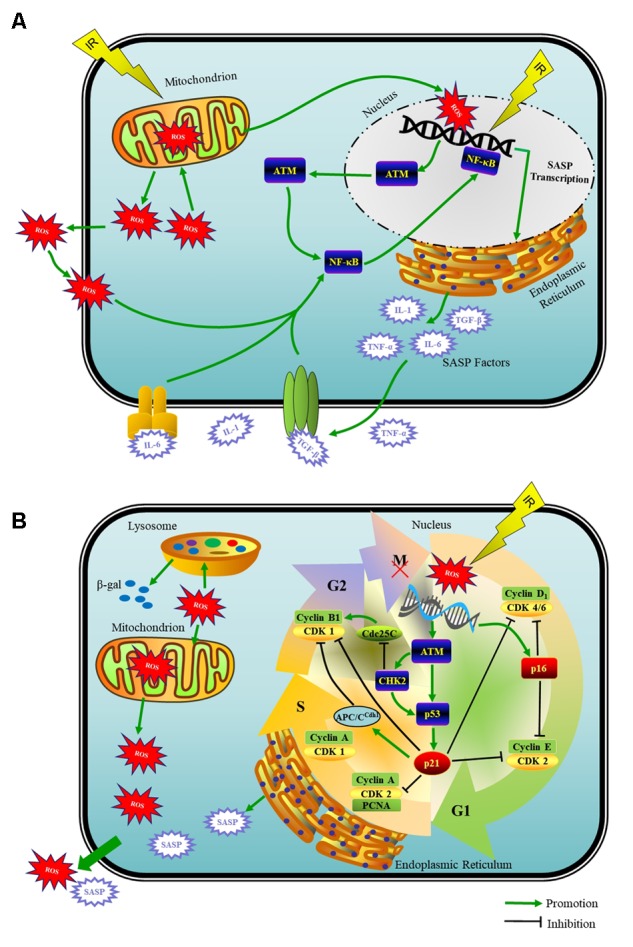
Schematic model of the DDR that induces a mitosis bypass and cellular senescence in response to IR. **(A)** When irreparable DNA damage initiates, cell cycle can get interrupted by G2 arrest for long time, which is followed by mitotic bypass into G1 phase with replicated DNA and culminates in cellular senescence. ATM- p53- p21, ROS produced by mitochondria, SASP factors and cyclin-CDK complexes are pivots of this senescence progress. **(B)** The recruitment of ATM to DSBs activates the NF-κB signaling that induces SASP expression including IL-1α/β, IL-6, TGF-β, and TNF-α et al. With potent autocrine and paracrine activities, SASP factors are co-opted to affect surrounding cells.

Physiologically, ROS are a by product of normal oxygen metabolism. Under damage from IR, mitochondria with energy deposited produce ROS in large quantities that are able to break the fine-tuned equilibrium between the oxidative and antioxidative processes (Chen et al., 2007). Specifically, in case of IR-induced DNA damage, mitochondria function as the main producer of ROS as well as one of the victims of ROS-induced oxidative damage. This damage to mitochondria *per se* further elevates the ROS generation, and therefore establishes a vicious cycle that maintains an ongoing DDR (Chan, 2006) (**Figure 1A**). Whether naturally or artificially produced, ROS are the main and persistent source of endogenous oxidative DNA damage in cells (Chen et al., 2007). ROS also impair other biomacromolecules, such as proteins and lipids (Vaiserman et al., 2016). Additionally, ROS are one of the necessary factors to touch off the DNA damage in nearby unirradiated cells according to the theory of BSE (Prise and O’Sullivan, 2009).

Although it is a truism that age-related changes in cells are accompanied by decreased mitochondrial function but upregulation of ROS levels (Karanjawala and Lieber, 2004; Chen et al., 2007), a cause-effect relationship does exist between cellular ROS production and senescence. ROS in high concentration have been proved to establish irreversible cellular senescence *in vitro*, such as in the typical SNC model–a variety of diploid fibroblast cells (Chen et al., 1995; Passos et al., 2010; Noh et al., 2016; Shimura et al., 2016). Under *in vivo* conditions, total body irradiation-induced residual bone marrow injury was attributed to the ROS-induced senescence in mouse hematopoietic progenitor cells, which was significantly attenuated by the application of various antioxidants (Shao et al., 2014). Akt activation was related to the increase of intracellular ROS levels, possibly by potentiating the oxygen metabolism and inhibiting the FOXO transcription factors. On the contrary, Akt-deficiency increased the resistance to oxidative stress-induced senescence (Nogueira et al., 2008). During the p53-dependent senescence process in endothelial cells, Akt activation promoted the senescence-like phenotype partly mediated by intracellular ROS levels (Miyauchi et al., 2004).

## DNA Damage

Various types and levels of DNA damage provoke different cellular responses, one of which is for surviving achieved by the transient activation of cell cycle checkpoints coupled with DNA repair, and another for terminating cell proliferation of irreparable cells either by cell death programs or by cellular senescence progress (Speidel, 2015). For example, exposure of cardiovascular endothelial cells to a very high dose (>10 Gy) of IR induced apoptosis, while exposure to a moderate radiation dose (0.5–10 Gy) primarily induced senescence (Wang et al., 2016).

Reactive oxygen species can cause either SSBs or DSBs. A subset of SSBs may evolve into DSBs (Chen et al., 2007). DSBs are the most deleterious DNA lesions mainly repaired via NHEJ that is orchestrated by DNA-PKcs. For cells in S and G2 phase, HR constitutes the second pathway to repair DSBs. Sophisticated DDR network that responds to DSBs include the recruitment of certain proteins (e.g., 53BP1 and Rad17), particular modifications like histone phosphorylation (e.g., γH2AX), or both (e.g., p-ATM), and the subsequent events (e.g., the activation of p53-p21 and/or p16-Rb signaling) to counteract DNA damage effects (Costes et al., 2007). Following IR, these repair proteins and checkpoint proteins are recruited to DSB sites or nearby within seconds to minutes, and form the RIF, such as γH2AX (Costes et al., 2007; Chiolo et al., 2013).

There are mainly two types of foci: transient (with successful NHEJ) and persistent (with irreparable DSBs) (Sedelnikova et al., 2004). Typically, repairable DNA damage foci are often transient and disappear within 24 h. However, severe or irreparable DNA damage endures because of the relatively stable structures and leads to cell growth arrest and interleukin-6 (IL-6) secretion that is a critical controller of autocrine senescence (Rodier et al., 2011). Recently, it has been reported that in senescent cells that lacked the nuclear envelope, chromatin fragments were extruded from the nucleus into the cytoplasm and stained positive for γ-H2AX, which finally initiated SASP factors secretion and proinflammatory responses (Ivanov et al., 2013; Dou et al., 2017). Therefore, persistent RIF may serve as a biomarker for cellular senescence and survival risk in relation to radiation exposure and DNA repair deficiency (Nelson et al., 2012; Chiolo et al., 2013). Collectively, cumulative irreparable DSB lesions shared among SNCs and DDR may have a causal role in triggering cellular senescence (Sedelnikova et al., 2004; Chen et al., 2007; Correia-Melo et al., 2016).

## SASP Factors

Initiation and maintenance of SASP producing requires ATM and the downstream NF-κB-dependent transcriptional program as the master regulators but not the cell cycle arrest enforcers p53 and Rb (Coppe et al., 2008; Rodier et al., 2009; Ferrand et al., 2015). The recruitment of ATM to DSBs activates the NF-κB that induces proinflammatory gene expression including IL-1α/β, IL-6, TGF-β, TNF-α, fibroblast growth factor, hepatocyte growth factor, and matrix metalloproteinases et al., which belong to the SASP factors (Piret et al., 1999; Orjalo et al., 2009; Ferrand et al., 2015; Childs et al., 2017) (**Figure 1A**).

Loss of ATM or Checkpoint kinase 2 (CHK2) that phosphorylates and activates p53, was not only able to steer clear of the p53-dependent senescence-related growth arrest but also prevent p53-independent secretion of SASP factors (Bartkova et al., 2006; Xue et al., 2007; Rodier et al., 2011). Afterward, Liao et al. (2014) demonstrated that IR affected surrounding, non-irradiated cells through SASP factors involving the AMPK- and NF-κB signaling pathways.

This is reminiscent of the BSE of IR, which supports that irradiated cells transmit signals, including oxygen radicals and cytokines, to neighboring non-irradiated cells through cellular gap junctions and media, thus affecting the microenvironments (Hagelstrom et al., 2008; Nelson et al., 2012). DNA repair protein ATM is necessary to trigger the bystander signals in human cells exposed to IR (Hagelstrom et al., 2008). Emerging evidence has indicated that SASP manipulates BSE to establish senescence and reconstruct microenvironments *in vitro* and *in vivo* (Prise and O’Sullivan, 2009; Nelson et al., 2012). The multifunctional cytokine IL-1α has been identified as the key factor to positively regulate the expression and secretion of IL-6/IL-8 in senescent human fibroblasts, all of which together created a pro-inflammatory and pro-senescence milieu (Orjalo et al., 2009). Several studies showed that only following the establishment of persistent DNA damage signaling could the SASP occur and underscored the major impact of SASP on altering tissue microenvironments (Coppe et al., 2008; Rodier et al., 2009). With the application of IL-6 knockout mice, Marmary et al. (2016) found that long after radiation-induced DNA damage, sustained expression of IL-6 was required to reinforce cellular senescence.

Collectively, with potent autocrine and paracrine activities, SASP factors are co-opted to induce inflammation and fibrosis, attract immune cells, induce malignant phenotypes in SNCs themselves and nearby cells, alter tissue microenvironments, and result in aging and age-related diseases (Tchkonia et al., 2013; Childs et al., 2017; Martinez-Zamudio et al., 2017; Schafer et al., 2017).

## IR-Induced G2 Arrest

The phenomenon that IR-induced cellular senescence could be launched after G2 arrest has gained evidence from numerous publications (reviewed in Gire and Dulic, 2015). Decades ago, Maity et al. (1994) showed that exposing a wide variety of cells to IR resulted in a mitotic delay that involved several events in G1, G2 or S phase, and that the G2 arrest was observed in virtually all eukaryotic cells and occurred following high and low doses, even under 1 Gy. However, the G1 arrest was often absent, while the S phase delay was typically seen following higher doses (>5 Gy) (Maity et al., 1994). Muthna et al. (2010) also found that human dental pulp stem cells lacked a G1 checkpoint in response to IR, and were preferentially arrested in the G2 phase (6–20 Gy). Moreover, these cells mainly established senescent state rather than apoptosis exposed to 20 Gy (Muthna et al., 2010).

The length of G2 has been found to be correlated with radiosensitivity, since radioresistant cell lines experienced a much greater G2 delay than the sensitive lines (Maity et al., 1994). It is noteworthy that the reversibility of the G2-arrest cells is determined in a much shorter time period compared to G1 block following DNA damage (Krenning et al., 2014). Additionally, cells encountering damage in late G2 terminate their cell cycle faster than cells receiving damage in early G2 phase (Mullers et al., 2014).

The mechanisms underlying IR-induced G2 arrest again shed light on the central role of ATM in the initiation and maintenance of genomic instability. Being one of the earliest known responders to DNA damage and a classical component of NHEJ, the ATM signaling cascade is activated within minutes in response to a DNA damage alarm, and its protein kinase activity is rapidly enhanced with the ability to phosphorylate its downstream targets involved in DNA repair, checkpoint control and apoptosis processes, such as CHK2, p53 and p21, which ultimately induces the G2 arrest (Ye et al., 2013; Kang et al., 2017). Correia-Melo et al. (2016) exposed human MRC5 fibroblasts to X-ray irradiation (20 Gy) and treated them with the ATM inhibitor KU55933. The inhibition of ATM ameliorated senescence-related phenotype following the activation of DDR at different time points (Correia-Melo et al., 2016).

In the early DDR, ATM activates CHK2, which, by sequestrating dual-specificity phosphatase Cdc25C in the cytoplasm to inhibit the activation of CDK1, prevents the activation of cyclin B1-CDK1 complex, a key mitotic regulator (Lapenna and Giordano, 2009; Gire and Dulic, 2015) (**Figure 1B**). Thus, cells with DNA damage cannot enter mitosis but have to wait in G2 phase transiently for repairing. In p53-proficient cells, DNA damage induces rapid upregulation (within 30–60 min) and stabilization of p53, and meanwhile ATM phosphorylates p53 at serine15, both of which are well-established markers of DNA damage-induced p53 activation. If the DNA damage cannot be repaired perfectly, DDR will move to the late phase, where activated p53 pathway stabilizes the G2 arrest (Speidel, 2015). In p53-deficient cells, early DDR merely blocks the G2/M progression temporarily or fails to induce a cell cycle arrest in the G2 phase (Krenning et al., 2014; Gire and Dulic, 2015). Therefore, cancer cells, half of which have lost the function of p53, may reenter the cell cycle after prolonged arrest and give rise to tumor recurrence (Salmina et al., 2010; Gewirtz et al., 2016).

Activation of p53 at G2 phase leads to the significant upregulation of p21 in the late phase of DDR, which consolidates the G2 arrest in several ways (Johmura et al., 2014). High levels of p21 engender the nuclear sequestration of cyclin B1 and/or mediate the degradation of cyclin B1-CDK1 complexes through the premature activation of APC/C APC/C^Cdh1^, which marks the point-of-no-return and constitutes the first step toward an irreversible G2 arrest before mitosis initiation (Krenning et al., 2014; Mullers et al., 2014, 2017). Moreover, p21 also inhibits cyclin D1-CDK4/6 complexes. Since cyclin D1-CDK4/6 complexes phosphorylate and inactivate Rb family proteins, p21 can indirectly activate Rb family proteins to inhibit the E2F1-dependent expression of mitotic regulators, thus halting the G2/M progression and resulting in the irreversible G2 arrest of cell cycle (Gire and Dulic, 2015). While p16 does not participate in the initiation of G2 arrest, it plays a key role in the maintenance and irreversibility of senescence (Beausejour et al., 2003; Johmura et al., 2014; Wang et al., 2016).

## Mitotic Bypass

In the normal cell cycle, the levels of cyclin B1 protein rise from late S-phase and culminate at late G2. Cyclin B1 binds to CDK1 to form the cyclin B1-CDK1 complex that is named “mitosis-promoting factor” and serves as a key regulator of mitotic entry. Cyclin B1-CDK1 complex is activated by the activating dephosphorylation of CDK1 that is the target of phosphatase Cdc25C (Harashima et al., 2013). CDK1 activation at the onset of mitosis results from the concurrent inhibition of Wee1 and Myt1 and stimulation of the phosphatase Cdc25C. Cyclin B1-CDK1 complex *per se* can also activate Cdc25C (Takizawa and Morgan, 2000). When cells progress into mitotic (M) phase (including mitosis and cytokinesis), cyclin B1 is degraded instantly on the anaphase onset (Nakayama and Yamaguchi, 2013). The precisely regulated spatiotemporal pattern of cyclin B1-CDK1 activity is pivotal for the normal cell cycle, which is subject to multiple control steps.

The knowledge that IR-induced SNCs are typically arrested in G2 phase raises an intriguing question: how can these SNCs overexpress cyclin D1 that is supposed to appear in the G1 phase and drives G1/S progression? This phenomenon is termed “mitotic bypass”, where mitotic regulators, such as cyclin B1, are suppressed and degraded leading to a direct skip of the mitosis (Johmura et al., 2014). Different from mitotic bypass, mitotic catastrophe is a form of cell death that can manifest as apoptosis. Mitotic catastrophe results from deficient cell cycle checkpoints (the DNA structure checkpoints and the spindle assembly checkpoint) in combination with cellular damage (for instance after IR-induced DNA damage) and frequently occurs on the premature activation of CDK1. Cancer cells are particularly sensitive to the induction of mitotic catastrophe instead of cellular senescence because of lacking effective mechanisms for cell cycle arrest, such as the key regulator p53 (Castedo et al., 2002, 2004).

IR-induced long-term G2 arrest is accompanied by the sequential occurrence of the mitotic bypass, G1 phase entrance and cellular senescence establishment (Ye et al., 2013). A delicate study discovered that human diploid fibroblasts exposed to various senescence inducers underwent a mitotic bypass and then irreversibly entered the cell cycle arrest in the form of tetraploid cells with G1-phase features. Time-lapse live-cell imaging revealed that these senescent fibroblasts bypassed mitosis before growth arrest between 24 to 48 h after IR treatment (Johmura et al., 2014). Another study employing human choroidal malignant melanoma 92-1 cells exposed to 10 Gy X-rays, found that the percentage of tetraploid cells amounted to 79.4 ± 0.06% within 15 h after IR and also supported that senescence-related long-term G2 arrest was induced along with the mitotic bypass and the slip into the G1 phase (Ye et al., 2013). Although this study used malignant 92-1 cell line, its p53-p21 signaling controls the cell cycle in the same way as normal, non-transformed cells do, which could also demonstrate the theory of mitotic bypass.

Mitotic bypass, which results from p53-p21-dependent premature activation of APC/C^Cdh1^ and suppression of mitotic regulators, appears to be a sufficient and necessary condition for inducing senescence both *in vitro* and *in vivo* (Davoli et al., 2010; Johmura et al., 2014). In brief, DNA damage-induced p53 activation, even transient activation, leads to p53-dependent nuclear sequestration of mitotic regulators. Subsequently, p21 prematurely activates the APC/C^Cdh1^-mediated degradation of mitotic regulators and S/G2-specific markers in combination with the CHK2-mediated cyclin B1 inhibition, thus inducing the bypass of mitosis and a tetraploid (4N) cell population (**Figure 1B**). This tetraploid cell population is characterized by G1-phase features, such as negative geminin (absent only in G1 phase) and the accumulation of G1 cyclins (cyclin D1) that drive G1/S progression, and positive DNA replication factor as well as SA-β-gal (Ye et al., 2013; Gire and Dulic, 2015). Non-transformed cells that have generated a tetraploid condition from failure of mitotic events will be arrested in G1 phase, which is dependent on one or more pathways that activate p53-p21 and/or p16-Rb signaling to restrain the function of cyclin D1-CDK4/6 and cyclin E-CDK2 that drive G1/S transition, thus blocking the tetraploid cells out of S phase (Andreassen et al., 2001; Ganem and Pellman, 2007). Therefore, we reasonably hypothesize that the overexpression of cyclin D1 is a kind of compensatory mechanism for the G1/S block, which, unfortunately, has been proved to actually compromise DNA repair (Pagano et al., 1994). This can possibly explain why SNCs are equipped with overexpression of cyclin D1 as a persistent marker (Leontieva et al., 2013).

## Conclusion

Ionizing radiation-induced cellular senescence serves as a double-edged sword during cancer treatment process. However, radiation-induced toxicity to normal tissues has been closely connected with organ dysfunction. If we expect to avoid cellular senescence and tumorigenesis in exposed healthy tissues and cells, issues that have to be settled firstly are a thorough investigation of the underlying mechanisms. This review sheds light on the mainstream mechanisms and principles of IR-induced DDR and cellular senescence. Since the parts of DDR interacts with each other at multiple points, subverting one part may also attenuate the effects of others, thus effectively interfering with the development of cellular senescence. Referring to the available senolytic agents, the development and identification of new drugs aiming at this DDR signaling network holds great potential to selectively remove SNCs and treat IR-induced diseases.

## Author Contributions

ML: drafted and wrote the manuscript. YL: conceived the idea for the manuscript. YL and JX: provided critical analysis and language editing. All authors contributed to the writing and final approval of the manuscript.

## Conflict of Interest Statement

The authors declare that the research was conducted in the absence of any commercial or financial relationships that could be construed as a potential conflict of interest.

## Abbreviations

ATM: ataxia telangiectasia mutated
APC/C: anaphase-promoting complex/cyclosome
APC/C^Cdh1^: APC/C bound to Cdh1
BSE: bystander effect
Cdc25C: cell division cycle protein C
Cdh1: cell-cycle regulated activator of the APC
CDK1: cyclin-dependent kinase 1
CHK: Checkpoint kinase
DDR: DNA damage response
DNA-PKcs: DNA-dependent protein kinase catalytic subunit
DSBs: DNA double-strand breaks
FOXO: Forkhead box protein O
HR: homologous recombination repair
IR: ionizing radiation
NF-κB: nuclear factor κB
NHEJ: non-homologous end-joining
RIF: radiation-induced foci
ROS: reactive oxygen species
SASP: senescence-associated secretory phenotype
SA-β-gal: senescence-associated β-galactosidase
SNCs: senescent cells
SSBs: DNA single strand breaks
